# An International Investigation of Molar Incisor Hypomineralisation (iMIH) and Its Association with Dental Anomalies: Development of a Protocol

**DOI:** 10.3390/dj11050117

**Published:** 2023-04-28

**Authors:** Helen D. Rodd, Hani Nazzal, Clarissa Calil Bonifacio, Choe Wei Ruth, Felicity Crombie, Osama El Shahawy, Morenike Oluwatoyin Folayan, Karla Gambetta-Tessini, Ashima Goyal, Noren Hasmun, Ahmad I. Issa, Suhad Jundi, David J. Manton, Srinivasan Narasimhan, Samah Omar, Susan Parekh, Bamidele O Popoola, Mihiri Silva, Greig Taylor, Yang Qiyue Naomi

**Affiliations:** 1 Unit of Oral Health, Dentistry and Society, School of Clinical Dentistry, University of Sheffield, Sheffield S10 2TA, UK; 2 Department Clinical Oral Health Sciences, College of Dental Medicine, Qatar University, Doha P.O. Box 2713, Qatar; 3 Hamad Dental Centre, Hamad, Medical Corporation, Doha P.O. Box 3050, Qatar; 4 Academic Center for Dentistry (ACTA), Gustav Mahlerlaan, 3004 Amsterdam, The Netherlands; 5 National University Centre for Oral Health Singapore, Singapore 119085, Singapore; 6 Melbourne Dental School, University of Melbourne, Melbourne 3010, Australia; 7 Pediatric Dentistry Department, Cairo University, Cairo 12613, Egypt; 8 Department of Child Dental Health, Obafemi Awolowo University, Ile-Ife 22005, Nigeria; 9 Department of Oral Rehabilitation, Faculty of Dentistry, University of Talca, Talca 3460000, Maule, Chile; 10 Oral Health Sciences Centre, PGIMER, Chandigarh 160012, India; 11 Department of Oral Sciences, Faculty of Dentistry, University of Otago, Dunedin 9054, New Zealand; 12 Ministry of National Guard Health Affairs, King Abdulaziz Hospital, Riyadh 36428, Al Ahsa, Saudi Arabia; 13 Preventive Dentistry Department, Faculty of Dentistry, Jordan University of Science and Technology, Ar-Ramtha P.O. Box 3030, Jordan; 14 University Medical Centre Groningen, University of Groningen, 9713 GZ Groningen, The Netherlands; 15 Pediatric Dentistry Department, Loma Linda University School of Dentistry, Loma Linda, CA 92350, USA; 16 UCL Eastman Dental Institute, London WC1E 6DG, UK; 17 University College Hospital, Agodi, Ibadan 200285, Nigeria; 18 Murdoch Children’s Research Institute, Royal Children’s Hospital, Melbourne 3052, Australia; 19 School of Dental Sciences, Faculty of Medical Sciences, Newcastle University, Newcastle-upon-Tyne NE2 4AZ, UK; 20 Youth Preventive Services, Health Promotion Board, Singapore 179369, Singapore

**Keywords:** molar incisor hypomineralisation, dental anomalies, hypodontia, protocol, taurodontism, MIH index, molar hypomineralisation

## Abstract

Background: Molar incisor hypomineralisation (MIH) is a common disorder of tooth development, which has recently been found to be associated with a higher prevalence of hypodontia. The aim of this international multicentre study is to determine the association between MIH and other developmental anomalies in different populations. Methods: Investigators were trained and calibrated for the assessment of MIH and dental anomalies and ethical approvals obtained in each participating country. The study aimed to recruit 584 children with MIH and 584 children without MIH. Patients aged 7–16 years who attend specialist clinics will be invited to participate. Children will undergo a clinical examination to determine the presence and severity of MIH, using an established index. The presence of any other anomalies, affecting tooth number, morphology, or position, will be documented. Panoramic radiographs will be assessed for dental anomalies and the presence of third permanent molars. Statistical analysis, using a chi squared test and regression analysis, will be performed to determine any differences in dental anomaly prevalence between the MIH and non-MIH group and to determine any association between dental anomalies and patient characteristics. Conclusion: This large-scale study has the potential to improve understanding about MIH with benefits for patient management.

## 1. Introduction

### 1.1. Background and Rationale

One of the most common disorders affecting tooth formation is molar incisor hypomineralisation (MIH), a variable developmental hypomineralised defect of enamel involving the first permanent molars and often the incisors, with an estimated global prevalence of 13% [1]. The management of children with MIH presents numerous challenges relating to the sensitivity and post-eruptive breakdown of affected molars, poor enamel bonding characteristics, and psychosocial concerns about incisor opacities [2,3]. Despite its common occurrence, speculation still surrounds its aetiology, as it is likely that several systemic and genetic and/or epigenetic factors are involved [4]. Within paediatric dental practice, a wide variety of other anomalies in tooth development are also seen, namely, relating to tooth number, morphology, and eruption. The reported prevalence of each of these different dental anomalies is highly variable, depending on the diagnostic criteria employed, as well as the characteristics of the study population [5,6]. Some dental anomalies, notably tooth number, appear to have a gender predilection; supernumerary teeth are twice more common in males than females, whereas hypodontia is more common in females than males [5]. Studies have also reported potential ethnic variation in the presentation of certain developmental dental conditions, with a systematic review reporting significant worldwide disparity in hypodontia prevalence, ranging from 4.4% in Latin America/Caribbean to 13.4% in Africa [7]. Most studies appear to show no gender difference in MIH but, to date, differences according to ethnicity have not been fully explored [1].

From a clinical perspective, it is important to recognise the potential for more than one oral/dental anomaly to be present in the same patient [8,9,10,11,12,13,14]. For example, children with a cleft lip and/or palate have a significantly higher incidence of hypodontia, not only in the region of the cleft, but in the whole dentition [15]. Another example of anomaly co-occurrence relates to hypodontia and taurodontism; children with congenitally missing teeth appear to have a significantly higher prevalence of taurodontism (34.8%) than their unaffected peers (7.5%) [16]. The potential for other anomalies to occur in children with MIH has only recently been recognised. An observational study of 101 children with MIH, conducted in a UK dental hospital, showed that 29% of patients had an additional dental anomaly [17]. The key finding was of a high prevalence of hypodontia, which affected 12% of the MIH patients (16.1% of girls and 2.6% of boys). These findings sparked interest amongst the international paediatric dentistry community.

In view of the important clinical and biological implications of any co-existence between MIH and other disorders of dental development, it was proposed that a multicentre international study (iMIH) should be undertaken to explore this preliminary finding further. The collaborative study would have additional value in determining any country/ethnic-related variations in the presentation of MIH and other dental anomalies, using a standard diagnostic approach. This paper describes the protocol for the planned study, including details of investigator training and calibration. The protocol was prepared according to guidance provided in the SPIRIT 2013 Checklist [18].

### 1.2. Aim and Objectives

The overall aim of this international multicentric analytical cross-sectional study is to explore any association between MIH and other developmental dental anomalies.

The primary objective is to:

- compare the overall prevalence of a dental anomaly (specifically hypodontia) in the permanent dentition of children (seven to sixteen years-of-age) with and without MIH.

The secondary objectives are to:

- determine the prevalence of other developmental dental anomalies, including: microdont maxillary lateral incisors; dens invaginatus/evaginatus; double teeth; supernumerary teeth; infraocclusion of primary molars; hypomineralisation of second primary molars; ectopic (palatal) position of maxillary canines; ectopic/failed eruption of first permanent molars, and taurodontism of mandibular first permanent molars and investigate the influence of gender and ethnicity/country;
- measure children’s self-reported global evaluation of their oral health in relation to their dental condition.

### 1.3. Null Hypothesis

There will be no significant difference in the prevalence of dental anomalies in children with or without a diagnosis of MIH.

## 2. Materials and Methods

### 2.1. Overall Study Design

This will be a cross-sectional analytical study involving children with MIH and a comparison group of non-MIH-affected children.

### 2.2. Establishment of the Investigatory Team and Study Setting

Following the publication by Walshaw and colleagues (2020) [17], which first suggested an increase in hypodontia prevalence in children with MIH, several paediatric dentists with an interest in MIH formed a collaborative research group to explore this association further. Prof. Helen Rodd (UK) and Prof. Hani Nazzal (Qatar) took on the role of joint primary investigators for the study, and a core group (named co-authors on this paper) was established to develop the protocol through a series of online meetings (held during 2020/21). The final investigatory team involved 22 senior paediatric dentistry colleagues across 15 countries (Australia; Chile; Egypt; Jordan; India; Netherlands; New Zealand; Nigeria; Qatar; Saudi Arabia; Singapore; Sudan; United Arab Emirates; United Kingdom; United States of America). The study will take place at specialist paediatric dentistry clinics in both teaching hospitals and community settings.

### 2.3. Eligibility Criteria

Participants are children, aged seven to sixteen years-old, with and without MIH, who meet the inclusion criteria, and who are attendant for assessment or treatment in the participating clinics. To avoid bias, the comparison group will not be solely drawn from a pool of orthodontic patients or those primarily referred with a known diagnosis of dental anomalies.

Generic inclusion criteria—all participants (both MIH and comparison groups):

- No significant medical history (ASA ≤ 2), syndromic conditions, cleft lip and/or palate
- Have an existing (full) panoramic radiograph at recruitment or subsequently undergoes one for routine diagnostic purposes
- Child able to accept detailed clinical examination, radiographs, and photographs
- Parents and children are able to consent/assent to participate in the study and have a sufficient level of literacy/understanding to complete written consent forms (with support if necessary).

MIH-group specific inclusion criteria:

- Children diagnosed with MIH by a specialist in paediatric dentistry according to validated diagnostic criteria [19,20]

Comparison-group specific inclusion criteria:

- Children referred to the host centre for the management of any dental condition other than MIH
- MIH-group specific exclusion criteria:
- Children with an enamel defect that is not typical of MIH
- Comparison-group specific exclusion criteria
- Children with an atypical pattern of dental caries/extraction in their first permanent molars such that a possible diagnosis of MIH could not be excluded [19,20]

### 2.4. Interventions

This study does not involve any intervention, as such. Participants will receive routine care, as deemed appropriate by the responsible clinician.

### 2.5. Outcomes

- The primary outcome measure for this study is the presence or absence of MIH and its association with the presence of another dental anomaly (specifically hypodontia) in the participants. Details of the index used to score the enamel defect are described in Section 2.8 below.
- Secondary outcome measures for this study include the clinical and/or radiographic severity of MIH in relation to participants’ gender, ethnicity/country, and their global self-reported oral health, details of which are also provided in the subsequent Section 2.8.

### 2.6. Timeline and Participant Involvement

Recruitment of participants has commenced on a rolling basis in individual countries, according to the date on which ethical approval was obtained for that site. No change to the protocol was advised by any of the ethical committees. The first participant was recruited on 6 March 2022, and recruitment is scheduled to continue until 31 July 2023. The burden on the participants is minimal because, according to informed consent, participants simply undergo a clinical and radiographic examination as part of their normal scheduled appointment. No additional visits are required, and participants are only asked to answer two questions about their oral health status. It is expected that the study will be completed by December 2023.

### 2.7. Sample Size

Based on the prevalence estimates of hypodontia in MIH (0.12 [17]) and non-MIH children (0.064 [7]), a sample size of 1168 children (584 per group) would provide 90% power in detecting the statistically significant difference in the association between MIH and hypodontia. The alpha error was set at 5%. Each of the 15 participating countries would, therefore, aim to recruit approximately 80 children (40 MIH and 40 non-MIH). This sample size was calculated using the G Power software (Version 3.1.9.7).

### 2.8. Data Collection

A clinical record form (CRF) form was developed and piloted prior to commencement of the main study (Supplementary document S1). The following non-identifiable patient and clinical variables will be recorded for each participant:

#### 2.8.1. Participants’ Demographics

Age; gender; ethnicity/country as appropriate (White; Mixed/Multiple ethnic groups; Asian; Black; Arabic; and other minority ethnic groups [to specify]).

#### 2.8.2. Clinical Variables

- The primary dental diagnosis will be recorded for both MIH and comparison participants (e.g., the comparison group could include patients presenting with orthodontic concerns, dental caries, traumatic dental injury, oral pathology/oral medicine-related issues, tooth surface loss, periodontal condition, or no dental condition but medical/behavioural reason for referral).
- Any known close family history (siblings or parents) of enamel defects, or missing or extra teeth, will be documented.
- Any enamel defect will be recorded using the validated MIH index [19,20]. All permanent teeth and second primary molars will be included, with scores given according to the nature of the defect (MIH or non-MIH) and, for MIH teeth, the severity and extent of the defect will also be recorded.
- Any other anomalies detected clinically will be noted, including: microdontia of permanent maxillary lateral incisors, dens invaginatus/evaginatus, double teeth, erupted supernumerary teeth, infraocclusion of primary molars (categorised as >1 mm below occlusal level of adjacent tooth/teeth), and ectopic eruption of first permanent molars.

#### 2.8.3. Participants’ Perspectives

To ensure children’s views are also sought, participants will be invited to answer two global oral health-related quality of life (OHRQoL) questions, using a 5-point Likert response, from the widely used and validated Child Perceptions Questionnaire [21]. These questions (Box 1) will be included in the CRF for each participant and the investigator will invite the child to respond.

Box 1 **Global oral-related quality of life questions to be completed by the participants [21].**

Would you say the health of your teeth, lips, jaws and mouth is:

  □ Excellent
  □ Very good
  □ Good
  □ Fair
  □ Poor

How much does the condition of your teeth, lips, jaws or mouth affect your life overall?

  □ Not at all
  □ Very little
  □ Some
  □ A lot
  □ Very much

#### 2.8.4. Radiographic Characteristics

All participants must have a full panoramic radiograph of good diagnostic quality, as indicated for their routine care, and not taken solely for the purpose of this study. This can be obtained from the participant’s past dental records rather than needing to be taken at the time of recruitment as long as the radiograph was obtained when the patient was seven to sixteen years-of-age. The following variables will be recorded from the radiographic presentation.

- Presence of any developing third molars.
- Hypodontia of permanent dentition (excluding third molars).
- Presence of any supernumerary teeth (specifying number and location).
- Presence of ectopic maxillary canines—this can only be coded ‘yes’ if patient has also had an additional intra-oral radiographic view.
- Presence of ectopic first permanent molars (a positive result would include evidence of distal resorption of second primary molars even if the first permanent molars have erupted subsequently).
- Any other anomaly that was not detected clinically (e.g., double tooth, dens invaginatus).
- Any other findings of note (e.g., any anomalies seen in the primary dentition).

#### 2.8.5. Assessment of Taurodontism

For patients with mature apical development of their first permanent mandibular molars (10 years and older), an objective assessment of taurodontism will be carried out using digital measurements from panoramic radiographs. The technique and criteria, described by Seow and Lai (1989) [16], will be adopted. The ratio (taken from vertical measurements) of the crown and body: root will be determined. The following classification system will be used to determine the degree of any taurodontism:

- Crown/body:root ratio < 1.1 = normal
- Crown/body:root ratio 1.1–1.29 = hypotaurodontism
- Crown/body:root ratio 1.29–2.00 = mesotaurontism
- Crown/body:root ratio > 2.00 = hypertaurodontism

### 2.9. Clinical Photographs

Wherever possible, clinical images of the participants’ dentition (maxillary and mandibular occlusal views, anterior view, and lateral views) will be taken and securely stored electronically as part of the data archive, as well as to support future investigations.

### 2.10. Investigator Training and Calibration

Prior to the start of the study, training and calibration on the use of a validated MIH index, which is supported by the European Academy of Paediatric Dentistry, was delivered to the two PIs by one of the original developers of this index (Dr Aghareed Ghanim, Clinical Senior Fellow, University of Melbourne, Australia) [19,20]. Following that, an online training programme was produced by the PIs using the same 20 clinical cases from the original training manual [20]. All investigators were required to complete this training before commencing recruitment.

Training and calibration for the collection of other clinical and radiographic parameters was delivered by Prof. Helen Rodd (Unit of Oral Health, University of Sheffield, UK). This training involved two pre-recorded presentations: one describing the various dental anomalies under investigation and the second describing the methodology for measuring taurodontism.

Inter-examiner and intra-examiner agreement was assessed following this training; investigators repeated the scoring for the various paraments two weeks after the initial exercise. All clinicians involved in recording these parameters were required to reach acceptable levels of repeatability (Cohen Kappa coefficient between 0.61 and 1.00).

### 2.11. Data Management

Data will be stored at each participating centre, in accordance with local policies and regulations. In general, collated data will be anonymised, encrypted, and stored on password-protected computers. Each unit will be responsible for anonymised electronic data entry for their participants (using Microsoft Excel spreadsheet software) and will send the complete data set securely to Prof. Hani Nazzal and Dr. Srinivasan Narasimhan for data analysis. Only the PIs will have access to the final study dataset.

### 2.12. Statistical Methods

Descriptive statistics and frequency distributions will be used to present the demographic and clinical characteristics of the study population. The chi square test (or Fisher exact test) will be used to assess potential differences in the prevalence of dental anomalies (specifically hypodontia) in children with and without MIH. The *p* value for statistical significance will be set at <0.05. In addition, logistic regression will be employed to determine the odds ratio (OR) and 95% confidence interval (CI) for the association between MIH and hypodontia (or any specific dental anomaly), after adjusting for age, gender, ethnicity/country, and MIH severity.

### 2.13. Research Ethics and Governance

Each investigator was required to seek appropriate ethical and clinical governance approvals from their own institute/service. Confirmation of ethical approval from each site is mandatory for study participation and must be sent to the PI (HZ) to upload to a password-protected shared drive as evidence of this approval. To governance-assure the present publication, details for the ethical bodies providing approval for the two PIs are as follows: Prof. Helen Rodd received approval from the Health Research Authority and Health and Care Research Wales on 2/12/2021 (REC ref: 21/WA/0376), and Dr. Hani Nazzal received approval from the Medical Research Centre, Hamad Medical Corporation (Ref: MRC-01-21-438| on 25/09/2022. Additionally, the study protocol was registered with ClinicalTrial.gov (Identifier: NCT05812690), a resource provided by the U.S. National Library of Medicine, which allows free public and professional access.

A standard protocol, CRF, patient information sheets, and consent/assent forms (Supplementary document S1) will be provided by the PIs for adaptation by each participating country (including translation into the local language). Specific approval will be obtained to share the anonymised participant data outside of each country, according to each site’s local regulations. Written informed consent will be obtained for study participation from parents/guardians, and written informed assent will be obtained from child participants themselves, according to each site’s local regulations. This is considered a low-risk study, with no anticipated harm to research participants. The investigators have no financial or competing interests with respect to this study.

### 2.14. Dissemination Policy

Findings from this study will be disseminated through presentations at scientific meetings and peer-reviewed publications with agreement from the whole team. It is anticipated that multiple papers and research theses may arise from this programme of work. An equable and transparent protocol for authorship of any related publications was, therefore, agreed by the whole team prior to starting the study. A participant-friendly end of study report will also be produced, with the input of child participants, to ensure the findings are accessible to the wider public.

## 3. Preliminary Results

To date, 39 paediatric dentists from the participating countries have undertaken training and calibration for the use of the MIH index [19]. Overall, 79.5% (n = 31) achieved substantial agreement (or above) on their first attempt; 15.4% (n = 6) repeated the training and subsequently reached the required level of agreement. However, two participants have yet to repeat the training exercise and thus cannot contribute to data collection until calibrated to the required standard. Excellent intra-examiner agreement was found for scoring both enamel defect type and extent (mean k = 0.86 and k = 0.83 respectively). Inter-examiner agreement was slightly lower, with a mean score of k = 0.74 for defect type and k = 0.74 for its extent.

With respect to measuring the degree of taurodontism, 24 paediatric dentists, from the different participating countries, undertook the training and calibration exercise. Overall, 87.5% (n = 21) achieved substantial agreement (or above) on their first attempt; 12.5% (n = 3) are still to repeat this training before progressing to data collection. Intra-examiner repeatability was almost perfect, with a mean kappa score of 0.95. Inter-examiner repeatability was substantial, with a mean kappa score of 0.65. Lack of agreement between examiners was predominantly related to uncertainty as to whether a tooth could actually be assessed; in cases where the crown cusps were not clearly visible, some examiners felt that they could not accurately identify the necessary landmarks. Further clarification has now been provided to investigators.

At the time of writing (April 2023), recruitment had reached 831 children (395 MIH and 436 non-MIH) from all 15 participating countries.

## 4. Discussion

There are several challenges, as well as benefits, inherent to conducting research, such as that proposed across multiple international centres. The initial establishment of the project necessitated quite complex and prolonged research/governance application processes, as well as the preparation of legal contracts to allow data sharing between centres. It is paramount that the highest standards of patient safety and data protection are upheld, but investigators should not underestimate the time, and even the cost, that these necessary approvals may take. Notwithstanding these requirements, the experience of establishing this unique research group and developing a consensus protocol was extremely positive. Social media and availability of high-performance virtual communication platforms, no doubt driven by the global COVID-19 pandemic, greatly facilitated our ability to network remotely.

As stated earlier, all potential investigators were required to undertake training and calibration prior to patient recruitment and data collection. Adherence to standard diagnostic criteria and evidence of good inter- and intra-examiner agreement was considered essential, allowing reliable comparison of findings between sites. It was encouraging that substantial intra- and inter-examiner agreement was found for scoring both enamel defect type and extent. The MIH index appears to be a valid tool to undertake a standardised assessment of this common but complex condition by the international paediatric dentistry community [19]. Therefore, it offers great potential for use in multicentre research studies to further the understanding of MIH epidemiology. Intra- and inter-examiner repeatability was also very good for the assessment of taurodontism, suggesting that the methodology adopted was robust and simple to follow.

It is hoped that the findings from this study will better inform the everyday management of children with MIH by alerting the clinician to the possibility of other dental anomalies, thereby prompting close clinical and radiographic monitoring of dental development. The presence of an additional anomaly may have considerable impact on treatment decisions, particularly in relation to the management of compromised first permanent molars. In general, a restorative approach is advocated for hypomineralised first permanent molars with post-eruptive enamel breakdown and/or caries lesions [2]. However, in some situations where these teeth are deemed to have extremely poor prognosis, their extraction at the optimum stage of dental development or in coordination with orthodontic care may be the preferred option [22]. In such cases, it is imperative to ascertain whether all other permanent teeth are present (ideally including third permanent molars). Thus, the congenital absence of a second premolar in the same quadrant would have considerable impact on any decision to extract the first permanent molar. Other missing teeth, such as permanent lateral incisors and second molars, could also affect the decision to extract one or more compromised first permanent molars. Furthermore, if a hypomineralised first permanent molar with poor prognosis was found to have a taurodont morphology, any future endodontic treatment would be more challenging. A controlled study, exploring any association between the presence of MIH and hypodontia, in addition to other dental anomalies, is clearly warranted to better inform patient management. The study may also provide new insights into the aetiology of MIH if it is found to be associated with dental conditions known to be under genetic control (e.g., tooth number), It is difficult, however, to predict the findings from this study in terms of any potential association between MIH and other dental anomalies. Early findings from some participating centres in the present study do not support this hypothesis, whereas other preliminary observations suggest the converse.

A further justification for undertaking this study is the potential to correlate not just the presence of MIH, but the severity of the condition, particularly the status of the first permanent molars (in terms of tooth tissue loss and sensitivity) with other dental anomalies and the presence/absence of developing third permanent molars. This line of inquiry would give valuable clinical insights to aid diagnosis and treatment planning for children with MIH, with the unique opportunity of exploring any ethnic/country differences. Despite a rapidly growing evidence base on the epidemiology, aetiology, and presenting characteristics of MIH, no standardised study has ever been conducted across such diverse populations.

## 5. Conclusions

This large-scale study has the potential to improve understanding about MIH and has implications for the management of children with this complex condition.

## Data Availability

No new data were created or analyzed in this study. Data sharing is not applicable to this article.

## Supplementary Materials

The following supporting information can be downloaded at: https://www.mdpi.com/article/10.3390/dj11050117/s1. Document S1: supporting protocol materials including: child assent form and information leaflet; parent consent form and information leaflet; clinical record form for all data collected in the study.
