# Supplementary material for: An International Investigation of Molar Incisor Hypomineralisation (iMIH) and Its Association with Dental Anomalies: Development of a Protocol
Source: Dent J (Basel). 2023 Apr 28;11(5):117. doi: 10.3390/dj11050117 (PMC10217515; doi:10.3390/dj11050117)
Supplement: Supplementary file 1 [file dentistry-11-00117-s001.zip › dentistry-2339153-supplementary.pdf]

The University Of Sheffield.

I.D. Number:

Supplementary Document S1

Participant assent form (11-16 years)

Do children with 'weak' enamel have any other tooth differences?

Thank you for completing this sheet.

Name of young person to be involved in the research:

____

Please tick the box if you agree with the sentence:

1. I have read and understood the information sheet for this project and that I have had the opportunity to ask questions. ☐

2. I understand that joining in this study is up to me and I am free to stop being in the study at any time, without giving a reason and without my dental care being affected. ☐

3. I understand that any information will be used for research purposes only; including research publications and reports. My name will never be mentioned. ☐

4. I give my permission to be sent a summary of the study findings ☐

5. I agree to take part ☐

Name of Young Person	Date	Signature
Name Researcher	Date	Signature

One copy for parent/guardian, one for researcher

The University Of Sheffield.

Do children with 'weak' enamel have any other tooth differences?

Hello!
I am Helen Rodd and I am a children's dentist. I am doing a project at the University of Sheffield. This is me.

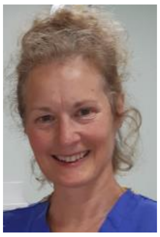

What is the study about and why we are doing it?
On our clinic we sometimes see children who have weak enamel because these teeth didn't grow properly. This condition is called **MIH** and the teeth can have brown or yellow or white marks on them (like in the pictures) and can be sensitive. We want to find out if children who have teeth like this also have other things that are different, like missing teeth or extra teeth. When we know more about this, we can make a better plan for other children in the future.

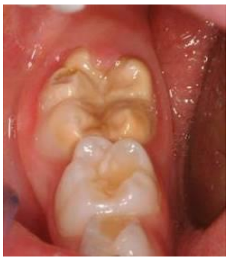

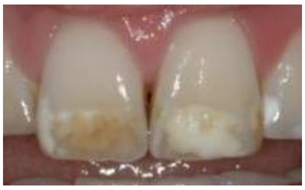

Who can join in?
We would like to see children aged 7-16 years who have marks on their teeth like in the pictures **AND** children who don't have any marks. Then we can compare the two groups. This is part of big study of around 1000 children and children in lots of different countries will be joining in too.

What will happen?
We will just do a check-up of your teeth with a mirror to see if you have any marks on your enamel. If you do, we will make a note of this. Our check-up will take about 10-15 minutes longer than a 'normal' one. We will also take photographs of your teeth (but not your face). If you have had an xray, like the one below, we will look at it to check all the teeth, even the ones that haven't grown yet. We will tell you what we find.

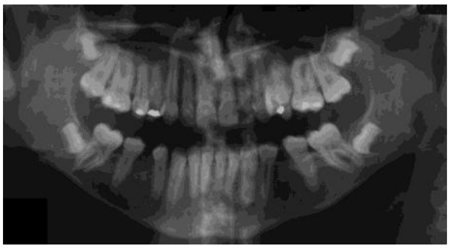

All the information about your teeth will be kept private. No one else will be able to look at it apart from the researchers working with me on this study. Your name or anything else that identifies you will not be used in any reports of the project. We will send you a letter at the end

of the project to tell you all about our findings, but your name won't be on any of the things we say.

How can I take part?
Taking part in this study is voluntary, which means it is your decision to take part or not. If you are interested in taking part, you can tell me, or one of the members of staff on the paediatric dentistry clinic. We can answer any questions you might have.

What if you don't want to join in anymore?
You may change your mind at any time without giving any reason. This will not affect any treatment or care you have in the future. There is no need to worry about stopping and that is fine with everybody here.

What if there is a problem?
We can't see anything going wrong during this project but if you or your parents feel unhappy about anything to do with the research, we are very happy to talk to you at any time. Please just contact us (or ask your parent/guardian to talk to us):

Professor Helen Rodd
Telephone 0114 271 7882 or email: h.d.rodd@sheffield.ac.uk OR
our Patient Advice and Liaison Service (PALS) STH.PALS@nhs.net

Thank you for reading this

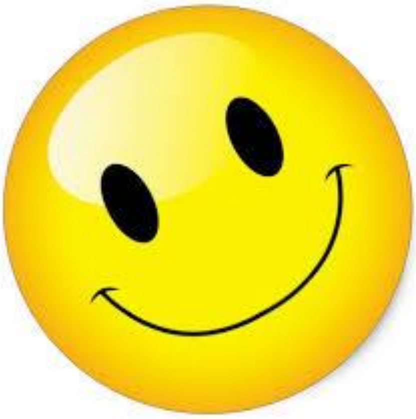

The University Of Sheffield.

I.D. Number:

Consent form for parents or guardians on behalf of a Child

Do children with 'weak' enamel have any other tooth differences?

Parent/guardian to

initial each

box

1. I confirm that I have read and understand the information sheet for the study (V1 31/12/2020). I have had the opportunity to consider the information, ask questions, and have had these answered satisfactorily.

2. I understand that my child's participation is voluntary and that I am free to withdraw my child at any time, without giving any reason, and without their dental care being affected.

3. I understand that sections of any of my child's dental notes may be reviewed by the researchers at the School of Clinical Dentistry, Sheffield University, where it is relevant to the research project. I also understand that sometimes research regulatory authorities may review my child's notes for monitoring and auditing purposes. I give permission for these individuals to have access to my child's dental records.

4. I agree to allow dental information about my child to be entered on a confidential computer database which will accessed only by the local project research team (Professor Rodd) and one other paediatric dentist (Dr Nazzal) who will be helping with the analysis of the international part of this study. I understand that personal details (including name and date of birth) will not be analysed, thereby maintaining my child's privacy.

5. I agree that digital photographs can be taken of my child's teeth and stored under a code name for analysis. The photographs will contain no recognisable facial features.

6. I agree dental radiographs (x-rays) taken for dental care may be retained and stored under a code name for analysis. Dental radiographs will not be taken for research purposes.

7. I agree that the information obtained for the purposes of this research study can be kept for 5-years before it will then be destroyed.

8. I would like a summary of the study's findings to be sent to me at the end of the study.

Parent/guardian name | Date | Signature

Name of researcher taking consent | Date | Signature

The University Of Sheffield.

Title of study:
Do children with 'weak' enamel have any other tooth differences?

Your child has been invited to take part in a study to find out whether children who have poorly mineralised (weak) enamel also have other dental differences (such as missing teeth, extra teeth, or teeth with different shapes or sizes). We are interested in an enamel condition called molar incisor hypomineralisation (MIH) which affects around 14% of children across the world. We are part of an international group of paediatric dentists who are researching MIH to see if it is associated with other developmental dental conditions and whether it is more common in certain ethnic groups. A small study of 100 British children recently found that children with MIH were more likely to have missing adult teeth. We would now like to carry out a larger study, to find out more about this possible link, so that we can provide the best possible dental care for children with MIH. But to be confident about our results, we also need to include children who don't have MIH, to see what proportion of them also have dental differences too.

This information leaflet gives you details of what will be involved if you decide to participate and also who to contact if you have any questions or would like to discuss any aspect of study. Please read the information leaflet carefully. We hope it helps you to make the decision whether to take part or not.

What is the study about?
This study is about a condition called Molar Incisor Hypomineralisation (MIH), which means the enamel of the adult first molars, and sometimes the incisors, has not formed properly. This condition may present as white chalky spots, or yellow-brown areas on teeth. The tooth can then 'crumble' or become decayed because it is weaker. The cause of this condition is still unclear, but it has been associated with problems around the time of birth and illnesses during early childhood.

This study will involve two groups of children: 1) children aged 7-16 years old who have been diagnosed by a specialist as having MIH and 2) children aged 7-16 years who do not have any enamel condition. The main aim of the study is to compare the frequency of dental differences (missing teeth, extra teeth, abnormal shape, size and position of teeth) in children with and without MIH, to see if differences are more common in children with MIH. We are also interested to know if the severity of MIH relates to the likelihood of any dental differences, and whether children from different ethnic groups are more or less likely to be affected. We hope this knowledge will help us understand more about MIH so we can provide the best management for children with this condition.

What is involved?
This study involves you and your child signing a consent form to confirm your child's participation in this study. Your child will have a check up to look at the overall condition and position of their teeth. The dentist will look very carefully at your child's first adult molars and incisors to see what the enamel looks like on these teeth. Your child's front and back teeth will be photographed, so we

can recheck the condition of the enamel, if necessary, at a later stage, and also to use some images in our future publications. Your child's face will not be photographed. Your child will be having an xray as part of their normal treatment (so it is not being taken just for the purposes of this research) and with your permission we will look at this radiograph to make a record of the shape and position of all your child's teeth, including whether or not they have any 'wisdom teeth' (third permanent molars). Your child's actual treatment will then be just the same as planned and will not be any different because they have taken part in this study. However, we think it will take 5-10 minutes longer to carry out our detailed dental check-up and photographs, that it would for a normal check-up.

We will send you a summary of the results at the end of the study which will be December 2023, if you are happy for us to do so.

Benefits, risks and safety

We do not anticipate any risks from this study as it involves just a simple check-up. The information that we obtain from this study will help us to understand more about MIH and other associated dental conditions. Your child will not benefit directly from taking part in the study, but the findings from the study will hopefully help us plan treatment better for other children with MIH.

Confidentiality and use of 'patient' data

All information gathered is confidential and nothing which identifies your child will be stored on a computer. All information from the research will be kept securely at the School of Clinical Dentistry, University of Sheffield and will be destroyed 5 years after the study has been completed. We will combine the data from our findings with findings from our paediatric dentistry colleagues around the world, so we will look at the results for around 1000 children. But no one will have access to the database apart from the research team, and there will be no personal data on the database. Your child will not be identified in any reports produced from the research project. The results will be shared at dental meetings and published in scientific journals. We have provided an additional sheet, if you wish to read further, about the use of patient data in health research as advised by the NHS Research Authority.

Participation

Taking part in this study is entirely voluntary. If you and your child decide to take part, you may change your mind and withdraw at any time without giving a reason. Your decision will not affect any ongoing or future dental care in any way.

At your dental check-up, if we think your child is eligible for the study, we will give you and your child and information sheet to take away. We will give you time to think if you would like to take part, and to ask any further questions you may have. If you would like to take part, you and your child will be asked to sign consent forms. You are both free to withdraw from the study at any time, without having to give a reason. If you don't wish to take part, that is fine, and your child's treatment will be just the same.

If you have any concerns or feel you would like to know more, please contact any of the following people:

Principal investigator - Professor Helen Rodd (School of Clinical Dentistry, University of Sheffield)

Telephone 0114 271 7882 or email: h.d.rodd@sheffield.ac.uk OR contact our Patient Advice and Liaison Service (PALS) STH.PALS@nhs.net

Thank you for reading this

NHS
Health Research Authority

This document explains how health researchers use information from patients. If you are asked to take part in research, you can ask what will happen in the study.

What is patient data?

When you go to your GP or hospital, the doctors and others looking after you will record information about your health. This will include your health problems, and the tests and treatment you have had. They might want to know about family history, if you smoke or what work you do. All this information that is recorded about you is called patient data or patient information.

When information about your health care joins together with information that can show who you are (like your name or NHS number) it is called identifiable patient information. It's important to all of us that this identifiable patient information is kept confidential to the patient and the people who need to know relevant bits of that information to look after the patient. There are special rules to keep confidential patient information safe and secure.

What sort of patient data does health and care research use?

There are lots of different types of health and care research.
If you take part in a clinical trial, researchers will be testing a medicine or other treatment. Or you may take part in a research study where you have some health tests or answer some questions. When you have agreed to take part in the study, the research team may look at your medical history and ask you questions to see if you are suitable for the study. During the study you may have blood tests or other health checks, and you may complete questionnaires. The research team will record this data in special forms and combine it with the information from everyone else in the study. This recorded information is research data.

In other types of research, you won’t need to do anything different, but the research team will be looking at some of your health records. This sort of research may use some data from your GP, hospital or central NHS records. Some research will combine these records with information from other places, like schools or social care. The information that the researcher collects from the health records is research data.

Why does health and care research use information from patients?

In clinical trials, the researchers are collecting data that will tell them whether one treatment is better or worse than other. The information they collect will show how safe a treatment is, or whether it is making a difference to your health. Different people can respond differently to a treatment. By collecting information from lots of people, researchers can use statistics to work out what effect a treatment is having.

Other types of research will collect data from lots of health records to look for patterns. It might be looking to see if any problems happen more in patients taking a medicine. Or to see if people who have screening tests are more likely to stay healthier.
Some research will use blood tests or samples along with information about the patient’s health. Researchers may be looking at changes in cells or chemicals due to a disease.
All research should only use the patient data that it really needs to do the research. You can ask what parts of your health records will be looked at.

How does research use patient data?

If you take part in some types of research, like clinical trials, some of the research team will need to know your name and contact details so they can contact you about your research appointments, or to send you questionnaires. Researchers must always make sure that as few people as possible can see this sort of information that can show who you are.
In lots of research, most of the research team will not need to know your name. In these cases, someone will remove your name from the research data and replace it with a code number. This is called coded data, or the technical term is pseudonymised data. For example, your blood test might be labelled with your code number instead of your name. It can be matched up with the rest of the data relating to you by the code number.
In other research, only the doctor copying the data from your health records will know your name. They will replace your name with a code number. They will also make sure that any other information that could show who you are is removed. For example, instead of using your date of birth they will give the research team your age. When there is no information that could show who you are, this is called anonymous data.

Where will my data go?

Sometimes your own doctor or care team will be involved in doing a research study. Often, they will be part of a bigger research team. This may involve other hospitals, or universities or companies developing new treatments. Sometimes parts of the research team will be in other countries. You can ask about where your data will go. You can also check whether the data they get will include information that could show who you are. Research teams in other countries must stick to the rules that the UK uses.

The bigger research team may include people who check the quality of the research. Regulators may also need to check the research. They will compare the recorded research data with your health records. They might read your health records through a secure internet connection or at the hospital or clinic. All the computers storing patient data must meet special security arrangements.

If you want to find out more about how companies develop and sell new medicines, the Association of the British Pharmaceutical Industry has information on its website.

What are my choices about my patient data?

• You can stop being part of a research study at any time, without giving a reason, but the research team will keep the research data about you that they already have. You can find out what would happen with your data before you agree to take part in a study.

• In some studies, once you have finished treatment the research team will continue to collect some information from your doctor or from central NHS records over a few months or years so the research team can track your health. If you do not want this to happen, you can say you want to stop any more information being collected.

• Researchers need to manage your records in specific ways for the research to be reliable. This means that they won't be able to let you see or change the data they hold about you. Research could go wrong if data is removed or changed.

What happens to my research data after the study?

Researchers must make sure they write the reports about the study in a way that no-one can work out that you took part in the study.

Once they have finished the study, the research team will keep the research data for several years, in case they need to check it. You can ask about who will keep it, whether it includes your name, and how long they will keep it.

Usually your hospital or GP where you are taking part in the study will keep a copy of the research data along with your name. The organisation running the research will usually only keep a coded copy of your research data, without your name included. This is kept so the results can be checked.

If you agree to take part in a research study, you may get the choice to give your research data from this study for future research. Sometimes this future research may use research data that has had your name and NHS number removed. Or it may use research data that could show who you are. You will be told what options there are. You will get details if your research data will be joined up with other information about you or your health, such as from your GP or social services. Once your details like your name or NHS number have been removed, other researchers won't be able to contact you to ask you about future research. Any information that could show who you are will be held safely with strict limits on who can access it.

You may also have the choice for the hospital or researchers to keep your contact details and some of your health information, so they can invite you to take part in future clinical trials or other studies. Your data will not be used to sell you anything. It will not be given to other organisations or companies except for research.

Will the use of my data meet GDPR rules?

GDPR stands for the General Data Protection Regulation. In the UK we follow the GDPR rules and have a law called the Data Protection Act. All research using patient data must follow UK laws and rules.

Universities, NHS organisations and companies may use patient data to do research to make health and care better. When companies do research to develop new treatments, they need to be able to prove that they need to use patient data for the research, and that they need to do the research to develop new treatments. In legal terms this means that they have a 'legitimate interest' in using patient data.

Universities and the NHS are funded from taxes and they are expected to do research as part of their job. They still need to be able to prove that they need to use patient data for the research. In

legal terms this means that they use patient data as part of ‘a task in the public interest’. If they could do the research without using patient data they would not be allowed to get your data. Researchers must show that their research takes account of the views of patients and ordinary members of the public. They must also show how they protect the privacy of the people who take part. An NHS research ethics committee checks this before the research starts.

What if I don't want my patient data used for research?

You will have a choice about taking part in a clinical trial testing a treatment. If you choose not to take part, that is fine. In most cases you will also have a choice about your patient data being used for other types of research. There are two cases where this might not happen:

1. When the research is using anonymous information. Because it’s anonymous, the research team don’t know whose data it is and can’t ask you.
2. When it would not be possible for the research team to ask everyone. This would usually be because of the number of people who would have to be contacted. Sometimes it will be because the research could be biased if some people chose not to agree. In this case a special NHS group will check that the reasons are valid. You can opt-out of your data being used for this sort of research. You can ask your GP about opting-out, or you can find out more.

Who can I contact if I have a complaint?

If you want to complain about how researchers have handled your information, you should contact the research team. If you are not happy after that, you can contact the Data Protection Officer. The research team can give you details of the right Data Protection Officer.
If you are not happy with their response or believe they are processing your data in a way that is not right or lawful, you can complain to the Information Commissioner’s Office (ICO) (www.ico.org.uk or 0303 123 1113).

Participant ID ☐☐☐-☐☐

Data collection sheet

MIH group ☐ Comparison non MIH group ☐

An international study of molar incisor hypomineralisation and its association with dental anomalies

Patient details

1. Date of this assessment: ………………………………………. (day/month/year)
2. Patient's age at this assessment: Years……… …Months………….
3. Gender: ☐Male ☐Female
4. Ethnicity: ☐White ☐Black ☐Asian ☐Arabic
   ☐Mixed/Multiple ethnic group (to specify): ……………………………………
   ☐ Other minority ethnic group (to specify): ……………………………………
5. Primary dental reason for referral (tick all that apply):
   ☐Caries ☐ Dental anomaly including MIH (specify)…………………………………………………
   ☐Oral pathology/oral medicine ☐Orthodontic need
   ☐Period condition ☐Tooth surface loss ☐Trauma
   ☐Other diagnosis (specify) ……………………………………………………………
6. Is there any **clear** family history of?
   ☐Hypodontia ☐ Supernumerary teeth
   ☐MIH ☐Enamel defects other than MIH, please specify ……………………………
   ☐Other anomalies (specify) ……………………………………………………………………

Patient input: ask the child to answer the following two questions & tick response given:

7. Would you say the health of your teeth, lips, jaws and mouth is:
   - ☐ Excellent
   - ☐ Very good
   - ☐ Good
   - ☐ Fair
   - ☐ Poor

   Option to record (in child's own words) reason/s for responding 'fair/poor'

8. How much does the condition of your teeth, lips, jaws or mouth affect your life overall?
   - ☐ Not at all
   - ☐ Very little
   - ☐ Some
   - ☐ A lot
   - ☐ Very much

   Option to record (in child's own words) reason/s for responding a 'lot/very much '

Clinical exam: circle all <u>erupted</u> teeth & code each tooth and surface using MIH index

	UPPER RIGHT		55	54	53	52	51	61	62	63	64	65	UPPER LEFT	
**Surface**	17	16	15	14	13	12	11	21	22	23	24	25	26	27
Buccal (labial)														
Occlusal (incisal)														
Palatal														

TURN OVER PAGE

	LOWER RIGHT		85	84	83	82	81	71	72	73	74	75	LOWER LEFT	
**Surface**	47	46	45	44	43	42	41	31	32	33	34	35	36	37
Buccal (labial)														
Occlusal (incisal)														
Lingual														

Following a clinical AND radiographic exam: enter code/s for each anomaly seen, circle tooth to indicate which tooth/site is affected (leave blank if no anomalies)

**UPPER RIGHT**	**Tooth**		55	54	53	52	51	61	62	63	64	65	**UPPER LEFT**	
	Anomaly code													
**Tooth**	17	16	15	14	13	12	11	21	22	23	24	25	26	27
Anomaly code														
**LOWER RIGHT**	**Tooth**		85	84	83	82	81	71	72	73	74	75	**LOWER LEFT**	
	Anomaly code													
**Tooth**	47	46	45	44	43	42	41	31	32	33	34	35	36	37
Anomaly code														

Record any additional clinical or radiographic findings of note

Presence of 8s: from radiographic findings circle any 8s seen to be developing

18	28
48	38

Degree of taurodontism: provide code (1-4) for crown:root ratio following measurements of lower first permanent molars. Code 5 for not assessed.

46		36	
